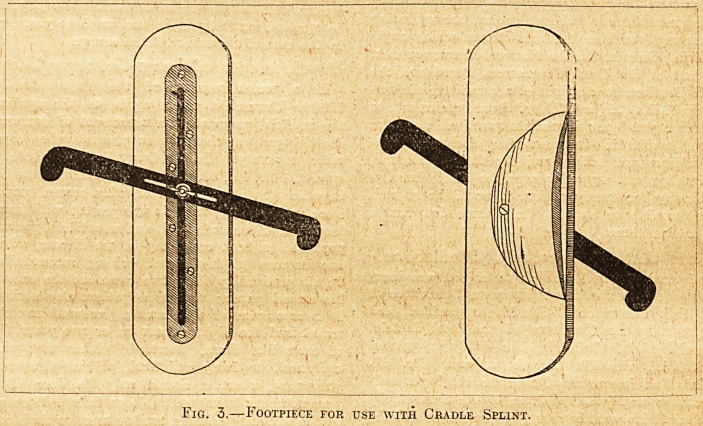# Gun-Shot Fractures of the Femur

**Published:** 1919-07-05

**Authors:** Ernest W. Hey Groves


### GUN-SHOT FRACTURES OF THE FEMUR.

By ERNEST W. HEY GROVES, M.S., F.R.C.S., MAJOR, R.A.M.C.

No class of case has occasioned more difficulty in the war, none has received more attention, and in none has systematic study produced so much improvement in results, as that of gun-shot fractures of the femur.

The knowledge and enthusiasm of Sir Robert Jones was fortunately at the service of the nation, and he was able to influence the administrative authorities, whilst he already possessed the entire confidence of the medical profession, in organising this matter. He made us conversant with the use of the Thomas knee splint, as the readiest method of dealing with a fractured femur, and one especially suitable for gun-shot injuries, because the thigh is slung from the side bars of the splint, and can be reached for dressing purposes without disturbing the bone. This splint will probably remain the officially recognised standard method, and it stands unrivalled for treatment during transport.

But the majority of those who have worked at this matter have recognised that in its original form the Thomas system is subject to various drawbacks and limitations. The chief of these are that it keeps the knee straight and makes it stiff; that however carefully the leg is drawn to full length and tied to the end of the splint, constant readjustment is necessary in order to maintain this; that the pressure of the ring of the splint against the buttock is painful and galling; that if the ring fits exactly, it is apt to constrict the thigh should the latter swell; that if the ring is made large it presses against the anus and genital organs; that the extension by adhesive plaster gives way after a few weeks, and in the renewal the fracture is disturbed, and that long application of plaster or glue is apt to make the skin sore, whilst it prevents access to the limb for massage.

All these difficulties have been overcome by various systems, some of which continue to employ the Thomas splint in a modified manner and others dispense with it.

### The Balkan Splint and Sinclair's Methods.

In the last Balkan war, under conditions which made the provision of special appliances very difficult, it was found that by hanging the leg to a beam above the bed, and by tying an extending weight to it, the best results were obtained which could be expected in the circumstances. With various modifications and elaborations the "Balkan beam" has proved of inestimable value in the present war.

The following is an apparatus which practically fulfils all requirements. A bar of wood, three inches square in section runs from the middle of the head of the bed to a point about 6 feet above one corner of the foot of the bed, where it is supported by a leaning beam, inclined outwards into the ward, so as to allow any weight hanging from it to hang clear. Pulley-wheels are attached to both the overhead bar and to that at the foot: these serve to sling up the leg from the bed, and to attach the extending weights. Any case received from the Front on a Thomas splint in which the position and condition of the limb are satisfactory is provided with this Balkan beam. The leg is slung off the bed by two cords, which are tied to the ring and lower ends of the splint. The cords may be merely tied to the beam, or may be made to run over pulleys and attached to weights so adjusted as to just counterbalance the weight of the leg and the splint. A third cord is tied to the foot of the splint, and this, taken over a pulley on the upright, is attached to a 15-lb. weight in order to produce extension, pulling the leg and the splint and releasing the buttock from pressure. Directly this has been done the patient experiences great relief, because the extension upon which fixation of the fracture depends then becomes constant and unvarying, quite apart from the adjustment of the splint, and also because the ring of the splint has its pressure taken away from the buttocks and perineum.

Major Sinclair has elaborated this principle by a series of most ingenious devices. The bed is surrounded by a framework of wood forming a veritable scaffolding, and this bears various pulleys, by which both counterpoise and extension can be maintained. In one of his appliances, designed for wounds high up in the thigh or buttocks, the patient lies with his head and body slung in a hammock,

Fig. 1.—Fracture of the Femur put up in Long Cradle Splint with Transfixion Extension.

**Treatment of Gun-Shot Fractures.**—(*continued*).

and each leg is slung in a separate net to beams. By this means any part of the under surface of the leg, buttock, or back can be exposed for dressing without disturbing the fracture, the position of which is maintained by weight extension attached by glued gauze strips. The draw-back to Sinclair's methods is the demands they make upon the ward space.

PEARSON'S BED.

Another apparatus is that invented by Major Maurice Pearson, and is one which, after some years of trial, was officially adopted by the War Office and supplied by them in large numbers some weeks after the war was over. The main principle is that the mattress and bedding can be readily removed from under the thigh, so as to leave the leg or buttocks free for dressing or nursing. The bed is about one foot higher than the usual hospital bed, and causes some difficulty to short nurses. Its lower end has telescopic legs, so that the foot of the bed can be raised by 18 in., or less, for the purpose of affording counter extension. Two overhead bars, one upright at the head and two at the foot, and one cross piece between the lower uprights, all made of metal tubing, are provided, in order to sling up one or both legs and to provide any required degree of abduction.

The mattress is made in three sections, and rests upon cross strips of canvas, one of which can be released in a moment by undoing two metal catches. This "quick-release" strip is placed under the smallest mattress section, and beneath the wounded thigh and buttock. The whole leg is kept on a Thomas splint, which is suspended to one of the overhead bars. Extension of the leg is made by a weight and cord, attached to the leg by means of an "ice-tongs" calliper, the points of which grip the lower end of the femur. A hinged frame is attached to the Thomas splint, opposite the knee joint, and to this is slung the lower portion of the limb, which can thus be bent daily without moving the broken thigh.

THE WIRE CRADLE SPLINTS.

These were designed by the author in the early stages of the war, and used first on a large scale in the general hospitals at Egypt. The cradle splint is a self-contained apparatus on which the leg is slung by means of flannel or rubber bands, and by means of which full extension can be obtained by the use of a weight and pulley attached to the framework of the splint itself. The extending weight takes its pull from a steel pin, which is usually driven through the upper end of the tibia, but which in certain cases transfixes the lower end of the femur. Counter-extension is secured by a padded band passed round the sound thigh and tied to the head of the bed. As the broken leg is pulled downwards, and the sound side of the pelvis is pulled upwards, the effect produced is a tilting of the pelvis and an abduction of the broken limb, a matter of great importance in dealing with fractures high up in the femur. The leg itself lies with the hip and knee bent, and this position is not only the most comfortable, but it is that which gives the best results in obtaining correct alignment of the fragments (see fig. 1).

Great care must be taken from the earliest days to secure correct position of the foot and great toe. If these are allowed to drop, a troublesome stiffness is liable to occur which will prevent the patient from putting the foot to the ground when he begins to walk. The foot must be slung up so that both the ankle and great toe joints are dorsi-flexed (*i.e.*, bent towards the knee). The sling which performs this office can conveniently be tied to the ends of the transfixion pin.

Usually 15 to 20 lb. weight will be required at first to pull the thigh to its normal length. After a few days it will be found that the leg is a little longer than its fellow, and the weight should then be reduced by one quarter or one third. The actual weight used is 5 lb. or 10 lb., a single solid iron weight. On the top bar of the extension uprights, and on the claw which takes hold of the transfixion pin, two pulley-wheels are running. If the cord bearing the weight be wound round all four pulleys, there is a fourfold multiplication of the extending force, and this can be reduced to threefold, double or single, by using three, two, or only one pulley-wheel. Thus, with the 5 lb. weight, an extension force of 5, 10, 15 or 20 lb. can be produced by the number of turns which the cord takes round the pulleys, without change of the weight itself (see fig. 2).

FIG. 2.—FRACTURE OF THE LEG BONES PUT UP IN "SHORT CRADLE SPLINT."

The whole limb lies comfortably slung in the cradle, exposed for massage or for dressing. When the latter is done, one or more slings are removed, rubber slings attached to the frame by paper clips being employed opposite to the wounds. Dressing is kept in place under the limb by the pressure of the slings, and over it, by tucking the ends of the dressing between the leg and the sling on each side. This makes the application and removal of the dressing a very simple matter, and one quite independent of the use of any bandage.

The mattress of the bed is of the biscuit pattern, the

**Treatment of Gun-Shot Fractures.**—(*continued*).

middle section being omitted and replaced by a ring air-cushion, on which the buttocks rest. For nursing purposes the cushion is replaced by a bed-pan, and for dressing high wounds it is replaced by a sandbag of suitable thickness placed under the buttock on the sound side.

After the first few days, when the patient has become accustomed to the splint, and when any inflammation of the wound has subsided, daily movements of the knee and ankle are undertaken. Proper execution of the knee movements does more than anything else to secure rapid functional recovery of the leg after the leg has been properly extended. The knee is steadied in its cradle by grasping it with one hand; the weight is then removed by taking off the claw hooked on to the transfixion pin, and with the other hand the foot is gently raised until the knee joint is fully extended. At first this need only be done for one complete movement each day. After about ten days greater amplitude should be given to the movements by taking away the slings below the calf and dropping the heel on to the bed before raising it to the knee level. Massage and active movements are carried out according to the rate of recovery of the fracture, and should always be ordered when early union has occurred. One advantage of the cradle splint is that it can be applied to any standard hospital bed, and does not require any special extra appliances. It does not take up any ward space outside the bed.

### Functional Recovery.

It cannot be too strongly insisted that the ultimate object of treating a fractured femur is to produce a good walking leg, and not merely a pretty $x$-ray picture. This object of proper functional recovery must be borne in mind from the very outset, because it is easier and much quicker to prevent the loss of function than to secure its recovery when once it is lost.

The three great causes which tend to make a man with a broken thigh a cripple are a deformed bone, a stiff knee, and wasted muscles, and a fourth minor cause is a dropped foot. Deformity of bone is prevented by adequate weight extension in the correct direction; the stiff knee is prevented by keeping the knee bent during splintage, and by moving it once every day; muscle-wasting cannot always be prevented if severe septic absorption has occurred, but otherwise it can be lessened by massage, controlled active movements of the knee, and by Faradic stimulation of the muscle groups of the thigh.

The patient should be got up as soon as possible—*i.e.*, when the bone has so far united as to make further displacement unlikely. When he first gets up he must wear either a walking calliper splint or an outside metal thigh splint securely attached to the pelvis above and to the lower end of the thigh below. Some such appliance is of the utmost importance in the case of fractures high up in the femur, because there is a great tendency to external angulation of the bone after this injury if a preventive splint is not worn. Directly a patient can walk a little without splints he must be taught to walk properly. Many a man limps badly long after a fractured thigh for the sole reason that he has acquired a bad habit which has never been corrected.

### The Importance of Segregation of Cases with Trained Team Work.

It may be a matter of surprise that no attempt has been made in describing the above three systems of treating fractured femurs to compare and contrast them, with the object of drawing a conclusion that one is better than the others. But I have done this with a set purpose, because, although I have a preference for the system which I have myself worked out, yet I am greatly impressed with the fact that certain administrative principles are much more important than the variety of appliance which is employed. These principles are those of segregation of fractured femurs in special hospitals or special wards, the continuity of treatment of the cases by surgeons and nurses accustomed to them, and the training of a team of surgeons, assistants, nurses, and masseuses who work together on a given plan understood by all. The introduction of these principles in the later years of the war has done more, I believe, than anything else to improve the results obtained. So long as cases were kept a few weeks in France, where the staff delayed adjustment of the fracture till transferred to England, and the staff in England assumed that adjustment had been done in France, and so long as cases were sent about from general

Fig. 3.—Footpiece for use with Cradle Splint.

**Treatment of Gun-Shot Fractures.**—(*continued*).

hospital to auxiliary by the administrative authorities, just so long were the results bad in the main and a discredit alike to the surgical profession and the Army Medical Service. But directly special hospitals were set apart both in France and England for fractured femurs, and cases kept in these until the men could walk, and teams trained to a correct method and a high ideal, then results began to be good, and it has been shown by workers like Major Pearson that under these conditions a large proportion of cases can be restored to perfect functional recovery. One is driven to ask whether this experience is not one in which civil surgery can learn a lesson from military.

It would certainly be of great advantage to the country if in all large cities the fractures and injuries of joints were brought together in special departments, the minor cases to be treated as out-patients, and severe cases as in-patients. Teams of surgeons, nurses, masseuses, and students should deal with these cases, and they should be kept under treatment and observation until they are as perfectly restored as the nature of their injury will permit.

At present the tendency in civil hospitals is to regard fractures as a nuisance unless they can be subjected to some dramatic operation. Otherwise they are pushed out as soon as they can be moved, and no one feels responsibility for their after disability. This system requires to be improved in the interests of the injured people, of the community who are deprived of their workers, and of the teaching and advance of surgical principles. It is high time that abdominal surgery should cease to dominate the civil hospitals, and that these institutions should be extended so as to provide for and to teach the adequate treatment of injured limbs.

* We are indebted to the Bristol Centre of the College of Nursing for the above paper, and to the Oxford Medical Publications for the loan of the blocks.—ED. T.H.

## Figures and Tables

**Fig. 1. f1:**
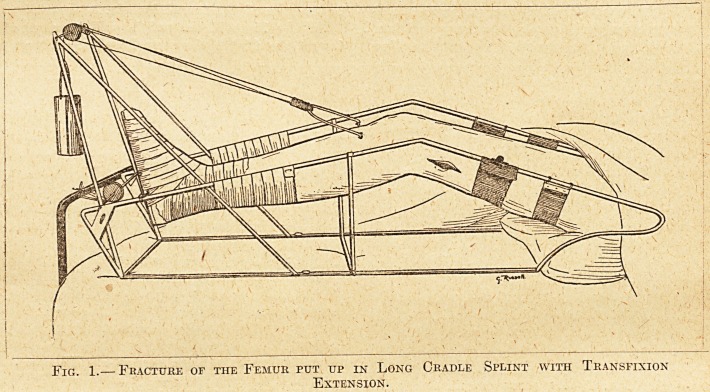


**Fig. 2. f2:**
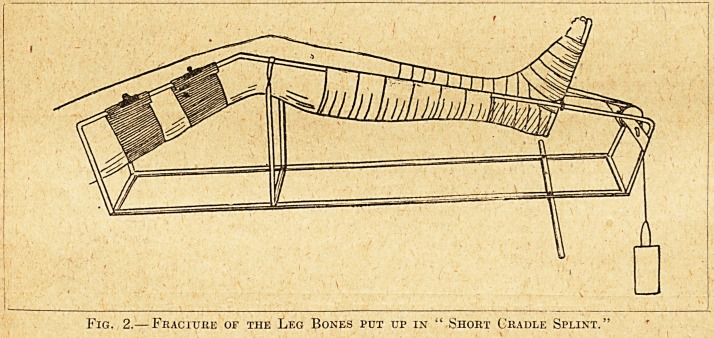


**Fig. 3. f3:**